# A method for the generation of large numbers of dendritic cells from CD34+ hematopoietic stem cells from cord blood

**DOI:** 10.1016/j.jim.2019.112703

**Published:** 2020-02

**Authors:** Nicole Bedke, Emily J. Swindle, Camelia Molnar, Patrick G. Holt, Deborah H. Strickland, Graham C. Roberts, Ruth Morris, Stephen T. Holgate, Donna E. Davies, Cornelia Blume

**Affiliations:** a Academic Unit of Clinical and Experimental Sciences, Faculty of Medicine, University of Southampton, Southampton, UK; b Telethon Institute for Child Health Research, Centre for Child Health Research, University of Western Australia, Perth, Australia

**Keywords:** Neonatal dendritic cells, CD34+ hematopoietic stem cells, Cord blood, Maturation, Poly(I:C), Lipopolysaccharide

## Abstract

Dendritic cells (DCs) play a central role in regulating innate and adaptive immune responses. It is well accepted that their regulatory functions change over the life course. In order to study DCs function during early life it is important to characterize the function of neonatal DCs. However, the availability of neonatal DCs is limited due to ethical reasons or relative small samples of cord blood making it difficult to perform large-scale experiments. Our aim was to establish a robust protocol for the generation of neonatal DCs from cord blood derived CD34+ hematopoietic stem cells. For the expansion of DC precursor cells we used a cytokine cocktail containing Flt-3 L, SCF, TPO, IL-3 and IL-6. The presence of IL-3 and IL-6 in the first 2 weeks of expansion culture was essential for the proliferation of DC precursor cells expressing CD14. After 4 weeks in culture, CD14+ precursor cells were selected and functional DCs were generated in the presence of GM-CSF and IL-4. Neonatal DCs were then stimulated with Poly(I:C) and LPS to mimic viral or bacterial infections, respectively. Poly(I:C) induced a higher expression of the maturation markers CD80, CD86 and CD40 compared to LPS. In line with literature data using cord blood DCs, our Poly(I:C) matured neonatal DCs cells showed a higher release of IL-12p70 compared to LPS matured neonatal DCs. Additionally, we demonstrated a higher release of IFN-γ, TNF-α, IL-1β and IL-6, but lower release of IL-10 in Poly(I:C) matured compared to LPS matured neonatal DCs derived from cord blood CD34+ hematopoietic stem cells. In summary, we established a robust protocol for the generation of large numbers of functional neonatal DCs. In line with previous studies, we showed that neonatal DCs generated form CD34+ cord blood progenitors have a higher inflammatory potential when exposed to viral than bacterial related stimuli.

## Highlights

• A robust protocol for the generation of high numbers of neonatal dendritic cells.
• IL-3 and IL-6 are crucial for the proliferation of cord blood CD34^+^ progenitors.
• Neonatal DCs have a higher inflammatory potential when exposed to viral stimuli.
• LPS induces higher release of IL-10 in neonatal DCs compared to Poly(I:C).

## 1 Introduction

Dendritic cells (DCs) act as sentinels of the immune system and therefore play a key role in triggering and priming immune responses to environmental agents. Through expression of receptors they sense infections and are able to link innate and adaptive immune response at epithelial interfaces (Hammad and Lambrecht, 2011). Through mediators including TSLP, OX40 ligand, GM-CSF, CCL20 and others released by the infected or damaged tissue, e.g. epithelial surfaces, DCs are recruited to the site of infection (Gill, 2012). In response, DCs are activated, migrate to draining lymph nodes and upregulate MHCII, co-activation receptors including CD80 and CD86 and secrete cytokines which can then instruct naive T-cells (Th0) to differentiate into Th1, Th2-cells, Treg or Th17 cells.

It is now well accepted that the characteristics of DCs change and mature during life (Willems et al., 2009; Basha et al., 2014; Agrawal et al., 2017). In order to understand the role of DCs over the life course, it becomes important to consider the source of DCs when studying their responses to environmental agents. In particular, due to the limited availability of neonatal DCs, it is challenging to investigate the characteristics of DCs at early life in detail. Hematopoietic stem cells (HSCs) have increasingly become an important tool to generate large numbers of immune cells of specific lineages due to their plasticity in being able to differentiate into a desired cell type depending on the cytokines they are exposed to. Specifically, methods to generate large numbers of DCs from CD34^+^ HSCs from cord blood (CB) using different cytokine cocktails have been developed, mainly for cancer immunotherapy (Balan et al., 2009; Harada et al., 2011; Plantinga et al., 2019). Our aim was to establish a robust protocol for obtaining large numbers of DCs from cord blood derived CD34^+^ HSCs. To assess functionality, we compared the responses of CB-derived neonatal DCs following exposure to the TLR3 ligand polyinosinic:polycytidylic acid (Poly(I:C)), an analogue of viral double-stranded RNA, and to the TLR4 ligand lipopolysaccharide (LPS), a component of the outer membrane of Gram-negative bacteria.

## 2 Materials and methods

### 2.1 Isolation of cord blood mononuclear cells (CBMCs)

CB samples were obtained following informed consent from mothers undergoing elective C-section (ethics code 07/Q1704/21 and 10/H0502/11) at the Princess Anne Maternity Hospital, Southampton and were processed within 30 min of collecting. CBMCs were isolated by means of Ficoll-Hypaque (GE Healthcare Life Sciences, Buckinghamshire, UK) gradient centrifugation and cryopreserved in 7.5% DMSO / 50% fetal bovine serum (FBS) until further use. CBMCs were thawed rapidly in a 37 °C water bath and media added. The cell suspension was centrifuged and incubated in the presence of DNase I solution (Stemcell Technologies, Grenoble, France) for 15 mins at room temperature. Cells were then counted and further used for magnetic isolation of CD34^+^ progenitor cells.

### 2.2 Isolation and expansion of CD34+ progenitor cells

CD34^+^ progenitor cells were obtained by magnetic isolation using the EasySep™ Human CD34 Positive selection kit (Stemcell Technologies, Grenoble, France) prior to plating in 24-well or 6-well plates at a cell density of 5 × 10^4^ cells per ml. Progenitor cells were cultured in IMDM (Life Technologies, Paisley, UK) containing 10% (*v*/v) heat-inactivated HI-FBS, 5 units/ml Penicillin G Sodium and 50 μg/ml Streptomycin sulphate, 0.1 mM β-mercaptoethanol (IMDM complete) with either 25 ng/ml Fms-related tyrosine kinase 3-Ligand (Flt3-L), 10 ng/ml Stem cell factor (SCF), 10 ng/ml Interleukin-3 (IL-3), 10 ng/ml IL-6 (IL-6) collectively called FS36 or 25 ng/ml Flt3-L, 10 ng/ml SCF, 10 ng/ml Thrombopoietin (TPO), collectively called FTS and incubated at 37 °C, 5% CO_2_ for a total of 28 days. Cells were cultured either for 1) 2 weeks in FS36, followed by 2 weeks in FTS; 2) 3 weeks in FS36, followed by 1 week in FTS; or 3) 4 weeks in FTS. After 7 days, cells were collected, centrifuged at 300 x*g*, 10 mins, RT, counted and re-seeded at 5 × 10^4^ cells per ml. At days 14 and 21, the cell densities were increased to 2 × 10^5^ cells per ml. At day 28, cells were seeded into a 6-well plate at 1 × 10^6^ cells per ml without FBS and incubated for 10 mins. Non-adherent cells were removed by washing twice with IMDM. Fresh media (IMDM complete) containing 20 ng/ml IL-4 and 50 ng/ml GM-CSF was then added to the adherent cells and cultured for another 5–6 days. Cells were fed every second day by semi-depletion with fresh IMDM complete containing GM-CSF and IL-4. All cytokines and growth factors were purchased from Peprotech (London, UK). For analysis of cell surface markers, cells were incubated and stained with fluorescent-conjugated antibodies to the cell surface markers CD1a, CD14, CD33 and CD34, (BD Biosciences, Oxford, UK) according to manufacturer's instructions. Whole cell populations were gated and analyzed by flow cytometry using a FACS Calibur. A Fixation and Dead Cell Discrimination Kit (Miltenyi Biotec, Surrey, UK, #130-091-163) was used according to the manufacturer's instructions to gate out populations of dead cells during analysis. The gating strategy is shown in Supplementary Fig. 1.

### 2.3 Stimulation and characterization of CBDCs

After GM-CSF and IL-4 treatments, cells were plated at a density of 5 × 10^5^ cells per ml in a 12-well plate. High-molecular weight polyinosinic:polycytidylic acid (PolyI:C, 1.5–8 kb)(Invivogen, Toulouse, France) or lipopolysaccharide (LPS) (Sigma, Dorset, UK) were added to cells at a concentration 10 μg/ml or 100–500 ng/ml respectively. Cells were incubated for 24 h at 37 °C, 5% CO_2_. For analysis of cell surface markers, cells were incubated and stained with fluorescent-conjugated antibodies to the cell surface markers CD1a, CD14, CD33, CD34, HLA-DR, CD40, CD80, CD86 (BD Biosciences, Oxford, UK) according to manufacturer's instructions. Whole cell populations were gated and analyzed by flow cytometry using a FACS Calibur. A Fixation and Dead Cell Discrimination Kit (MiltenyiBiotec) was used to gate out population of dead cells during analysis.

### 2.4 Cytokine assay

Cell supernatants were analyzed for secretion of human IFN-γ, IL-1β, IL-6, IL-10, IL-12p70 and TNF-α using a multi-array cytokines assay according to manufacturer's instructions (Meso Scale Discovery, Rockville, USA).

### 2.5 Statistical analysis

Statistical evaluation was performed using the software SigmaPlot 12.5. If not stated otherwise, related samples were analyzed for statistical significance using the Shapiro-Wilk test for normality followed by a paired Student's *t*-test or Wilcoxon Signed Rank test. Differences were regarded as significant when *P* ≤ .05. For multiple comparisons, ANOVA followed by Tukey test or the non-parametric Friedman followed by Dunn's was performed.

## 3 Results

### 3.1 Establishment of expansion cell cultures from cord blood CD34^+^ hematopoietic stem cells

**Fig. 1:**
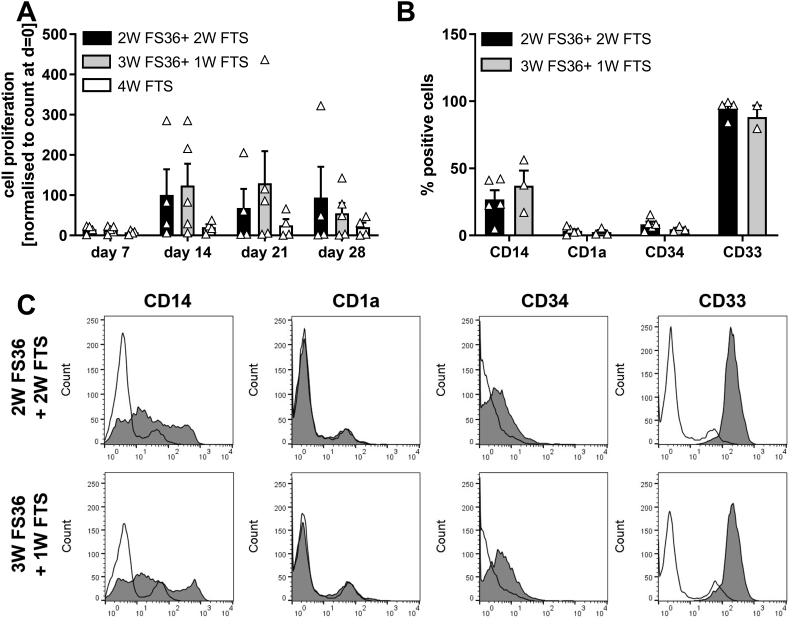
Expansion of cord blood CD34+ progenitor cells using different cytokine cocktails. A: proliferation of progenitor cells in the presence of different cytokine cocktails. B: Expression of surface markers after 28 days of culture. Mean ± SEM. *n* = 3–5 independent experiments each using different donors. C: Representative histogram overlays. (thin line: isotype control, grey infill: marker expression; 2 W FS36 + 2 W FTS: 2 weeks culture in Flt3-L, SCF, IL-3 and IL-6 followed by 2 weeks Flt3-L, TPO and SCF. 3 W FS36 + 1 W FTS: 3 weeks culture in Flt3-L, SCF, IL-3 and IL-6 followed by 1 weeks Flt3-L, TPO and SCF. 4 W FTS: 4 weeks Flt3-L, TPO and SCF.

Our aim was to establish a robust method to expand precursors of DCs from CB CD34+ HSCs for the generation of DCs that can be used for analyzing neonatal immune responses. Work published by Balan et al. (2009) used IMDM medium supplemented with a cytokine cocktail containing 25 ng/ml Flt3-L, 10 ng/ml TPO and 20 ng/ml SCF and 5% heat-inactivated autologous cord blood plasma. However, using these conditions in our experiments, the CB stem cells failed to survive beyond day 7. Therefore, we altered the culture conditions based on data from the literature (Bontkes et al., 2002). After purification of CB CD34^+^ cells by magnetic separation CD34^+^, cells were cultured for a total of 4 weeks in either medium containing Flt3-L, SCF, IL-3 and IL-6 (FS36) for 2 weeks followed by 2 weeks days in Flt3-L, TPO and SCF (FTS) or were cultured for 3 weeks in FS36 medium followed by 1 week in FTS. As a comparison, cells were also cultured for 4 weeks in FTS alone. We obtained the highest number of CD34^+^ derived progenitor cells when cultures were grown in the presence of IL-3 and IL-6 (Fig. 1A), which is in line with previous observations (Bontkes et al., 2002). The ability of CD34^+^ HSCs to proliferate was highest within the first 2 weeks of culture after which cell numbers plateaued or decreased slightly (Fig. 1A). This correlated with a decreased expression of the surface marker CD34 indicating that cells had lost their pluripotency (Supplementary Fig. 2S). Cells cultured for 4 weeks in FTS showed very low proliferation demonstrating a key requirement for IL-3 and IL-6 for growth. After 4 weeks in culture the progenitor cells were further characterized by flow cytometry and both culture conditions showed comparable expression of cell-surface markers. The majority of cells were positive for the myeloid lineage marker CD33 (88–94%). Between 25 and 30% were positive for the monocyte-specific marker CD14 (25–30%), and a low percentage of cells were positive for the DC specific marker CD1a (2.5–3.5%) and the hematopoietic stem cell marker CD34 (4.7–8.3%) (Fig. 1B and C).

### 3.2 Generation of dendritic cells from expanded progenitor cells derived from cord blood CD34+ hematopoietic stem cells

**Fig. 2:**
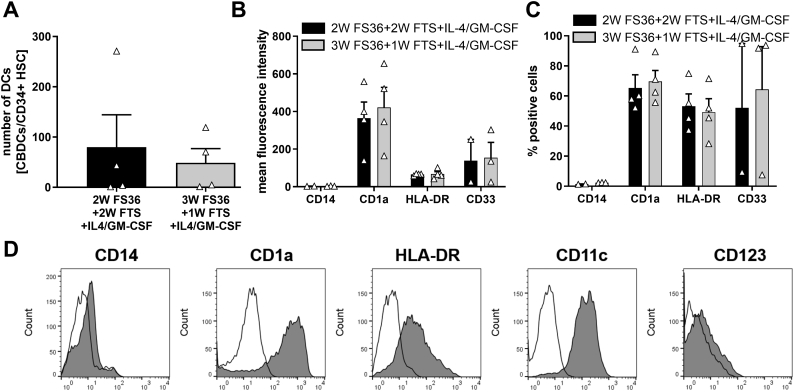
Expression of DC-specific surface markers of neonatal DCs generated from expanded CD34+ progenitor cells. After 4 weeks of expansion culture using cytokine cocktails detailed in Materials and Methods section, neonatal DCs were generated using IL-4 and GM-CSF. A: The number of DCs generated using the 2 different expansion culture conditions is expressed per CD34+ hematopoietic stem cell (HSC). B and C: Expression of surface markers was analyzed by flow cytometry. Mean fluorescence intensity (B) and % positive cells (C) of neonatal DCs generated from 2 different CD34+ cell expansion culture conditions. Mean ± SEM. *n* = 4 independent experiments using different donors. D: Representative histogram overlays of surface marker expression by neonatal DCs generated from 2 W FS36 + 2 W FTS expansion cultures. (thin line: isotype control, grey infill: marker expression).

After 28 days of expansion culture using either 2 W FS36 + 2 W FTS or 3 W FS36 + 1 W FTS, DC were generated from the progenitor cells following culture for 5–6 days in the presence of GM-CSF and IL-4. Use of 2 W FS36 + 2 W FTS or 3 W FS36 + 1 W FTS expansion culture resulted in a similar number of DCs (Fig. 2A). After culture for 5–6 days in GM-CSF and IL-4 neonatal DCs derived from either protocol expressed high levels of CD1a, a marker of DCs, HLA-DR and CD33, a marker of myeloid linage, whereas expression of CD14, a monocyte specific marker CD14, was negligible (2 W FS36 + 2 W FTS: 1.42 ± 0.14% positive cells, MFI 3.0 ± 0.69; 3 W FS36 + 1 W FTS: 2.42 ± 0.08% positive cells, MFI of 3.65 ± 0.68 (mean ± SEM)) (Fig. 2B-D). Over 60% of the cells were CD11c^+^HLA-DR^+^ (Supplementary Fig. 3S). This marker profile identifies the cells as DCs. There was no difference in the ability of the DC progenitor cells obtained from the two different expansion culture conditions to differentiate into DCs. These data demonstrate that high numbers of neonatal DCs can be generated from CD34^+^ HSC from cord blood.

### 3.3 Maturation of DCs

**Fig. 3:**
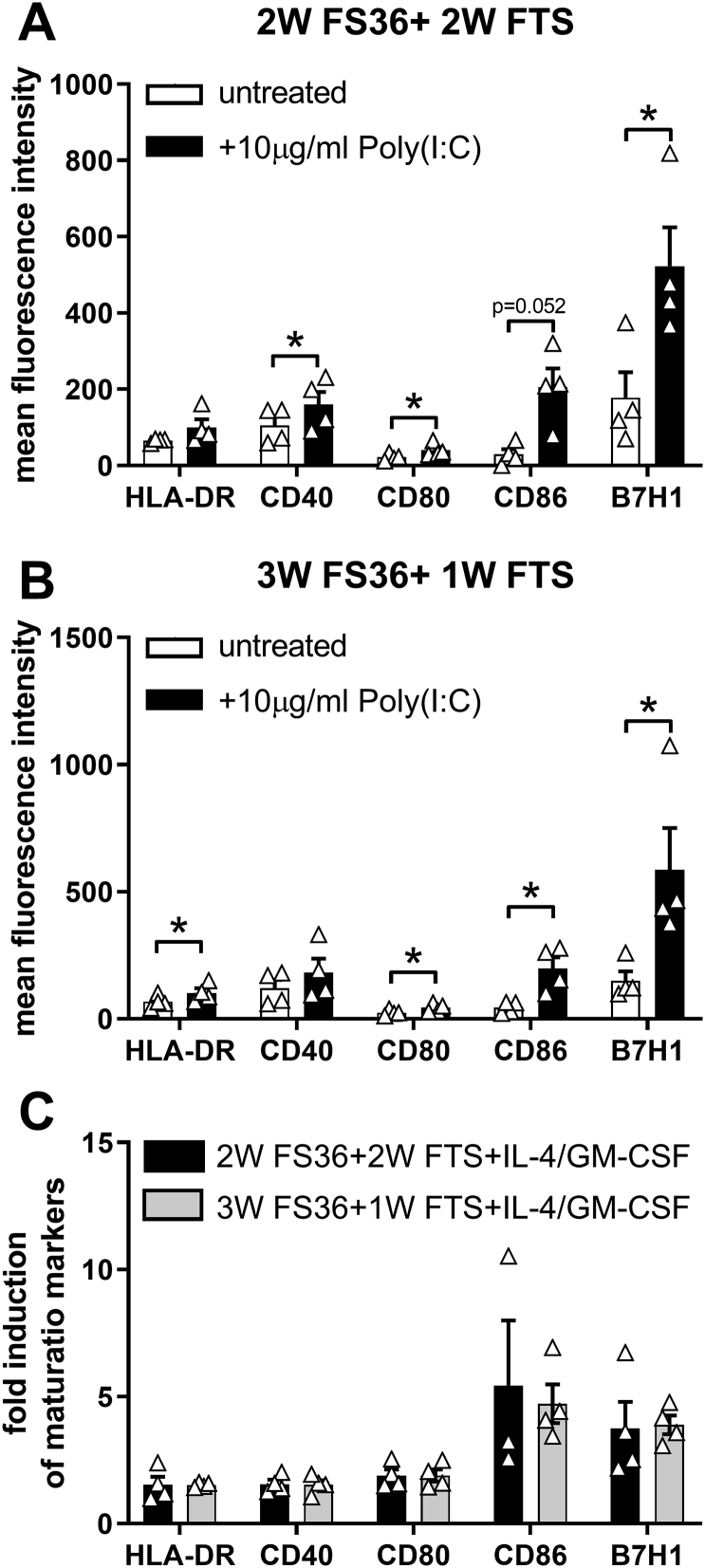
Expression of maturation markers by neonatal DCs. After expansion of CD34+ hematopoietic stem cells using 2 different cytokine cocktails and differentiation into DCs maturation was induced by incubation with Poly(I:C) for 24 h. Expression of surface markers on immature and mature DCs was analyzed by flow cytometry. A: Expression of maturation markers by neonatal DCs that have been generated from expansion cultures using 2 weeks Flt3-L, SCF, IL-3 and IL-6 followed by 2 weeks in Flt3-L, TPO and SCF (2 W FS36 + 2 W FTS). B: Expression of maturation markers by neonatal DCs generated from expansion cultures using 3 weeks Flt3-L, SCF, IL-3 and IL-6 followed by 1 weeks in Flt3-L, TPO and SCF (3 W FS36 + 1 W FTS). C: Fold induction of maturation markers by mature neonatal DCs normalised to the level of immature DCs generated from CD34+ expansion cultures using 2 different cytokine cocktails. Mean ± SEM. *n* = 4 independent experiments using different donors. *: *p* ≤ .05.

**Fig. 4:**
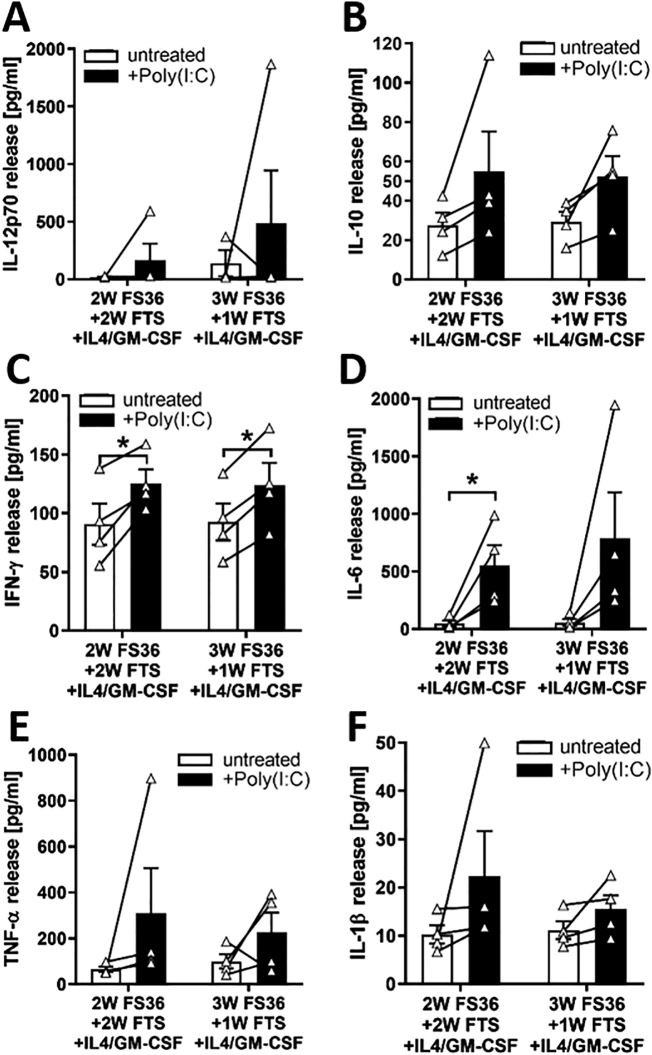
Release of cytokines by neonatal DCs after maturation. Neonatal DCs were generated from CD34+ progenitors using 2 different cytokine cocktails for expansion culture as described in the Method section. DCs were matured by incubation with 10 μg/ml Poly(I:C) for 24 h and the release of mediators determined in cell-free supernatants by multiplex assay. A: IL-12p70; B: IL-10; C: IFN-γ; D: IL-6; E: TNF-α; F: IL-1β. Mean ± SEM. n = 4 independent experiments using different donors. *: p ≤ .05.

Next we investigated whether the CB-derived neonatal DCs generated from precursors expanded using the 2 different culture conditions described above showed any differences in their ability to mature. Following exposure to the TLR3 ligand Poly(I:C) the expression levels of CD40, CD80 and B7H1 were significantly increased compared to the unexposed control in CBDCs generated from 2 W FS36 + 2 W FTS expansion cultures and the expression levels of HLA-DR, CD80, CD86 and B7H1 were significantly increased compared to the unexposed control in CBDCs generated from 3 W FS36 + 1 W FTS expansion cultures (Fig. 3). However, the fold induction of these surface markers in Poly(I:C) exposed CBDCs compared to the unexposed control was comparable between the two different expansion methods used for the DCs precursor cells. After maturation, DCs release a variety of cytokines that are able to modulate T cell function. Therefore, we compared the cytokine profile of mature CBDCs generated from precursors that have been expanded using the two different culture conditions. As shown in Fig. 4, mature CBDCs showed an increased release of IFN-γ, IL-1β, IL-6, IL-10, and TNF-α but only IFN-γ reached statistical significance. Release of IL-12p70 was unchanged. Furthermore, there was no difference between the culture conditions in the levels of cytokines generated. In summary, we did not observe any difference in the maturation of CBDCs derived from precursors that have been expanded using the two different expansion culture conditions.

### 3.4 TLR-dependent maturation of CBDCs

**Fig. 5:**
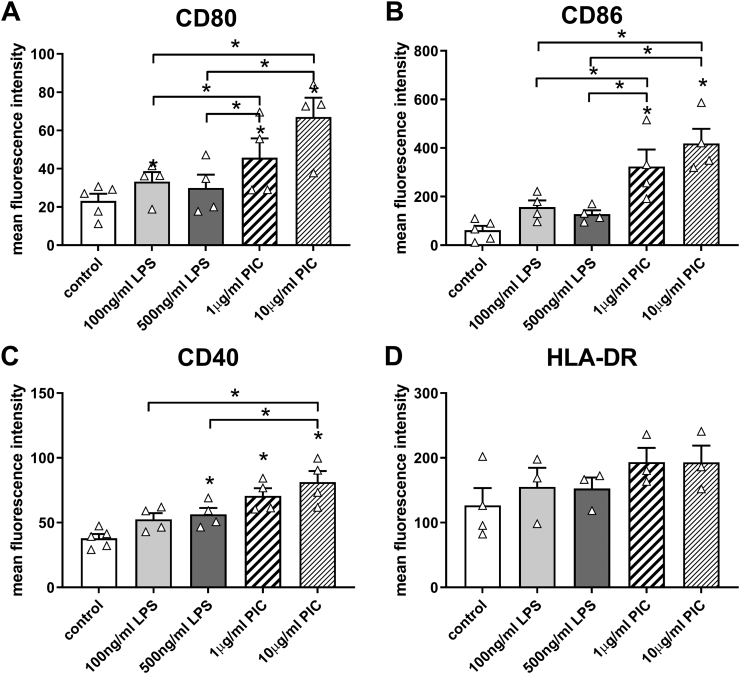
Expression of maturation markers by neonatal DCs varies with different TLR activation. Neonatal DCs were incubated for 24 h with the TLR4 ligand LPS or the TLR3 ligand Poly(I:C) and the expression of surface markers analyzed by flow cytometry. A: CD80; B: CD86; C: CD40; D: HLA-DR. Results shown are means ± SEM, n = 4 independent experiments using different donors. *: p ≤ .05 (ANOVA followed by Tukey multiple comparisons test).

**Fig. 6:**
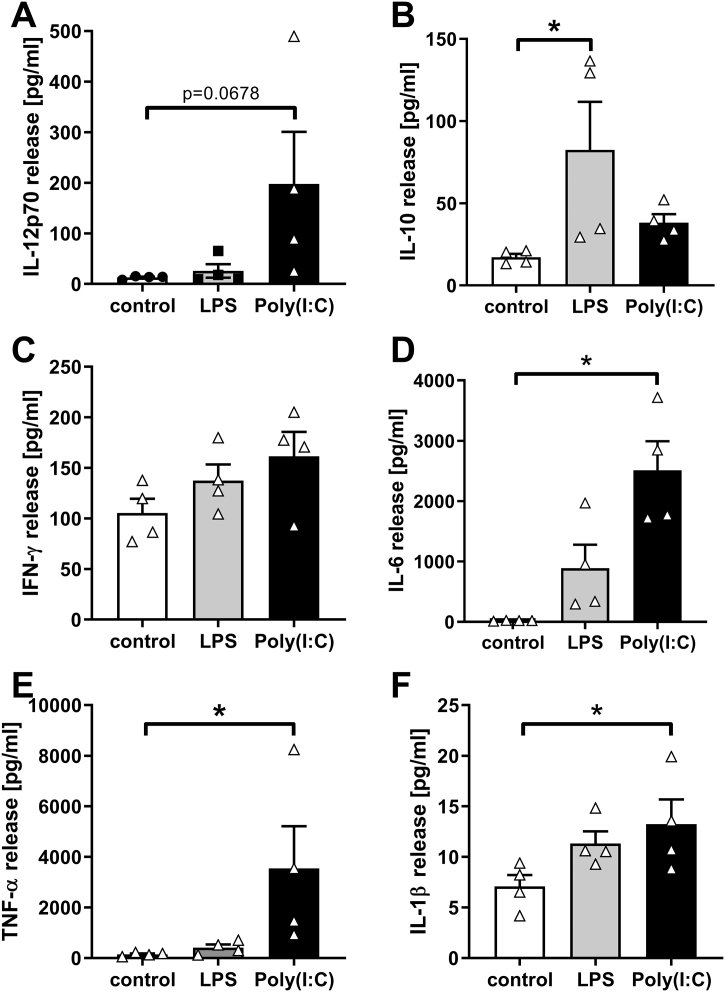
Differential release of cytokines after maturation of neonatal DCs with TLR3 or TLR4 ligands. After incubation with LPS (TLR4 ligand) or Poly(I:C) (TLR3 ligand) for 24 h the release of mediators was analyzed in the cell culture supernatants by multiplex assay. A: IL-12p70; B: IL-10; C: IFN-γ; D: IL-6; E: TNF-α; F: IL-1β. Results shown are means ± SEM, n = 4 independent experiments using different donors. *: p ≤ .05 compared to control (Friedman test followed by Dunn's multiple comparisons test).

In order to assess the potential of CBDCs to mature in response to different TLR stimuli we analyzed their response to the TLR4 agonist LPS and to the TLR3 agonist Poly(I:C). For this set of experiments, we used neonatal DCs that had been generated from 2 W FS36 + 2 W FTS expansion cultures since expansion was slightly higher using this culture condition and the ability of neonatal DCs to mature was not affected by the expansion culture condition. As shown in Fig. 5, Poly(I:C) at 10 μg/ml induced a significantly higher expression of the maturation markers CD80, CD86, and CD40 compared to LPS used at 100 μg/ml and 500 μg/ml. Expression of HLA-DR showed no significant change. Since 100 μg/ml LPS induced a slightly higher CD80 and CD86 expression than 500 μg/ml LPS, we used this concentration of LPS to analyze the cytokine release further. Additionally, we chose 10 μg/ml Poly(I:C) to mimic virally induced maturation as this concentration showed a significant effect on maturation marker expression by neonatal DCs compared to LPS, which mimics bacterial induced maturation. Interestingly, release of IL-10 was only significantly increased by LPS, whereas release of IL-6, TNF-α and IL-1β were only significantly increased by Poly(I:C) compared to the control (Fig. 6). These results suggest that neonatal DCs show a higher inflammatory potential when maturation is induced by virus-related compared to bacteria-related components.

## 4 Discussion

DCs play an important role in bridging the innate and adaptive immune response to infection. It is known that immune responses change over the life course (Simon et al., 2015) and DCs are thought to be centrally involved in the priming process. However, the availability of neonatal human DCs for functional in vitro studies is limited resulting in an unmet need for a source of large numbers of neonatal DCs that can be used in detailed mechanistic studies. Therefore, our aim was to establish a robust protocol for the generation of high numbers of DCs from CB-derived HSCs that have a more ‘naïve’ phenotype and can be used to investigate the characteristics of DCs in the context of early life. Previous studies have used CD34^+^ HSCs cultured in a cocktail of growth factors to expand precursors and drive their differentiation into DCs. While Chang et al. (2012) expanded cord blood derived CD34+ HSCs by using SCF only for expansion cultures, Harada et al. (2011) used a combination of GM-CSF and SCF. Arrighi et al. (1999) and Balan et al. (2009) expanded CD34+ HSC from cord blood using a cocktail of Flt3-L, TPO and SCF. Using this cocktail for expansion, we only observed a moderate expansion of DC precursor cells. Interestingly, when using heat-inactivated autologous serum instead of FCS as described by Balan et al. (2009), cells failed to survive for more than one week in culture. Therefore, we aimed to optimize the expansion culture conditions in order to increase the proliferation rate of CD34+ HSC from cord blood that would result in a high number of precursor cells that can be differentiated into functional DCs. We adapted an expansion protocol that was previously used for CD34+ HSC from adult peripheral blood, where CD34^+^ HSC using Flt3-L, TPO, SCF, IL-3 and IL-6 followed by treatment with GM-CSF and IL-4 to generate DCs (Bontkes et al., 2002). As previously reported, we observed the highest increase in precursor cells in the presence of IL-3 and IL-6, which made a large-scale experimental setup more feasible. Ultimately, we were able to differentiate myeloid DCs that were CD1a^+^CD11c^+^CD40^+^HLA-DR^+^ and CD14^−^CD123^−^, surface markers that are characteristic of human myeloid DCs (Baharom et al., 2017). Although it has been reported that DCs with high expression of CD11c can be obtained using only GM-CSF and SCF for expansion culture, these cells also expressed high levels of the monocyte marker CD14 (Harada et al., 2011). As the authors did not analyze expression of CD1a or CD123 it is difficult to compare the purity or functionality of the cells. Furthermore, it has been shown that CD34^+^ CD117^+^ cord blood cells are multipotent and able to develop into monocytes, granulocytes, B cells, pDCs, CD1c^+^ and CD141^+^ cDCs when co-cultured with stromal cells and Flt3-L, SCF and GM-CSF (Breton et al., 2015). This underlines the importance of the conditions used for expansion culture and differentiation which can determine the characteristics of the resulting cell populations. Further work will be required to fully characterize the surface marker expression for a in depth comparison of DC subtypes generated using our protocol. Previously, concerns have been raised about the prolonged presence of IL-3 and IL-6 in the expansion cultures leading to decreased levels of IL-12p70 and increased levels of IL-10 (Bontkes et al., 2002; Ebner et al., 2002). However, we did not observe any significant differences in the secretion of either cytokine from mature CBDCs generated from expansion cultures using IL-3 and IL-6 for the first two weeks or the first 3 weeks of expansion culture. A shorter GMP protocol, which has applicability in cancer immunotherapy, consisting of one week expansion culture with Flt3-L, SCF, IL-3 and IL-6 followed by one week differentiation into DCs using Flt3-L, SCF, GM-CSF and IL-4 has been used by Plantinga et al. (2019) to expand CD34+ stem cells into a population consisting of about 34% CD11c^+^HLA-DR^+^ DCs. In contrast, our method resulted in over 60% CD11c^+^HLA-DR^+^ DCs. Additionally, Plantinga et al. demonstrated that CB-derived DCs originate exclusively from CD115+ progenitor cells and resemble cDC2. In summary, we established a protocol that allows the generation of high numbers of neonatal DCs from CB-derived HSCs that can be used to analyze the DC immune response in an early life setting.

In our study, we analyzed the ability of CBDCs to respond to different stimuli. High molecular weight Poly(I:C), which is an analogue of double-stranded RNA that is produced during the replication cycle of certain viruses and has been shown to activate TLR3, RIG-I/MDA5 and PKR, caused a more Th1 biased cytokine profile with high IL-12, IFN-γ, TNF-α, IL-6 and IL-1β release. Using LPS to induce maturation, a bacterial membrane component of Gram-negative bacteria, we observed a significantly induced release of IL-10. These findings are consistent with previous studies using CB-monocyte derived DCs which showed that Poly(I:C) induced maturation resulted in higher IL-12p70 release compared to LPS (Goriely et al., 2001) and indicate that the immune responses specific for neonatal DCs are sustained in our model system.

Our observation of significantly increased IL-6 release from neonatal DCs during Poly(I:C) induced maturation, which mimics a viral infection, might have important modulatory functions during the polarization of naive T cells in early life. It has been shown that exogenous IL-6 in addition to TGFβ is an important factor during Th17 polarization (Swain et al., 2012; Martinez-Sanchez et al., 2018). While the role of Th17 cells in viral infections is not fully understood, there is evidence that Th17 cells can support neutrophil recruitment via release of IL-17 (Ye et al., 2001; Mukherjee et al., 2011). Additionally, Th17 cells are thought to play an important role in the anti-microbial host defense at mucosal surfaces (Rathore and Wang, 2016). In order to demonstrate the direct T cell stimulating ability of these DCs further experiments using DC-T cell co-cultures need to be performed. Through access to in vitro generated neonatal DCs, the mechanisms of innate and adaptive immune responses to viral and bacterial infections early in life can now be investigated in detail. This is of importance as this time in early life, described as the neonatal window of opportunity, is linked to the susceptibility for many immune disorders in later life (Torow and Hornef, 2017). In particular, this has the potential to identify the mechanistic link between early life infections and the risk of developing chronic diseases later in life. Furthermore, there is evidence that innate immune responses of neonates are influenced by maternal factors in utero. For example, Hrdy et al. (2014) showed a higher IL-12 gene expression in neonatal CB-DCs derived from allergic compared to non-allergic mothers. Interestingly, a reduced release of IL-6 was observed in CB-derived monocytes after stimulation with LPS in neonates with maternal allergy compared to non-allergic controls (Saghafian-Hedengren et al., 2008). However, further detailed mechanistic investigations are needed using neonatal DCs in order to establish a functional link infections early in life, IL-6 production, Th17 cells function and the risk of developing chronic diseases later in life. In conclusion, we present here a robust protocol for the generation of high numbers of neonatal DCs that are derived from CD34+ HSCs from CB that can be used for mechanistic studies of early life immune responses.

## Author disclosure statement

The authors declare no competing interests.

## Appendix A Supplementary data

Supplementary material Image 1
