# Supplementary material for: A method for the generation of large numbers of dendritic cells from CD34+ hematopoietic stem cells from cord blood
Source: J Immunol Methods. 2020 Feb;477:112703. doi: 10.1016/j.jim.2019.112703 (PMC6983936; doi:10.1016/j.jim.2019.112703)
Supplement: Supplementary file 1 — Supplementary material [file mmc1.docx]

**Supplementary Figures**

**A method for the generation of large numbers of dendritic cells from CD34+ hematopoietic stem cells from cord blood**

Nicole Bedke, PhD^1^, Emily J. Swindle, PhD^1^,Camelia Molnar, MSc,^1^,Patrick G. Holt, PhD^2^,Deborah H. Strickland, PhD^2^,Graham C. Roberts, DM^1^, Ruth Morris RSCN^1^, Stephen T. Holgate, DSc, MD,^1^ Donna E. Davies, PhD^1^, Cornelia Blume, PhD^1^

^1^: Academic Unit of Clinical and Experimental Sciences, Faculty of Medicine, University of Southampton, Southampton, UK

^2^: Telethon Institute for Child Health Research, Centre for Child Health Research, University of Western Australia, Perth, Australia


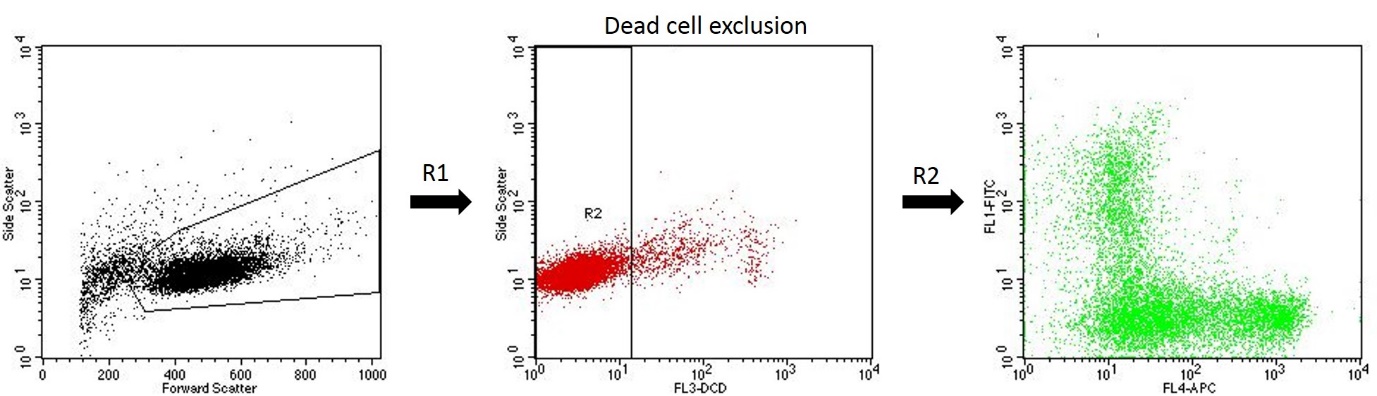


**Supplementary Figure 1S: Gating strategy of cord blood progenitor cells during expansion culture for the exclusion of dead cells.** Cells in R1 were plotted for the dead cell discriminator (DCD) in FL3 vs side scatter. Subsequently, DCD-negative cells in R2 were plotted for FL1 (FITC), FL2 (PE) or FL4 (APC) depending on the used labelled antibodies.


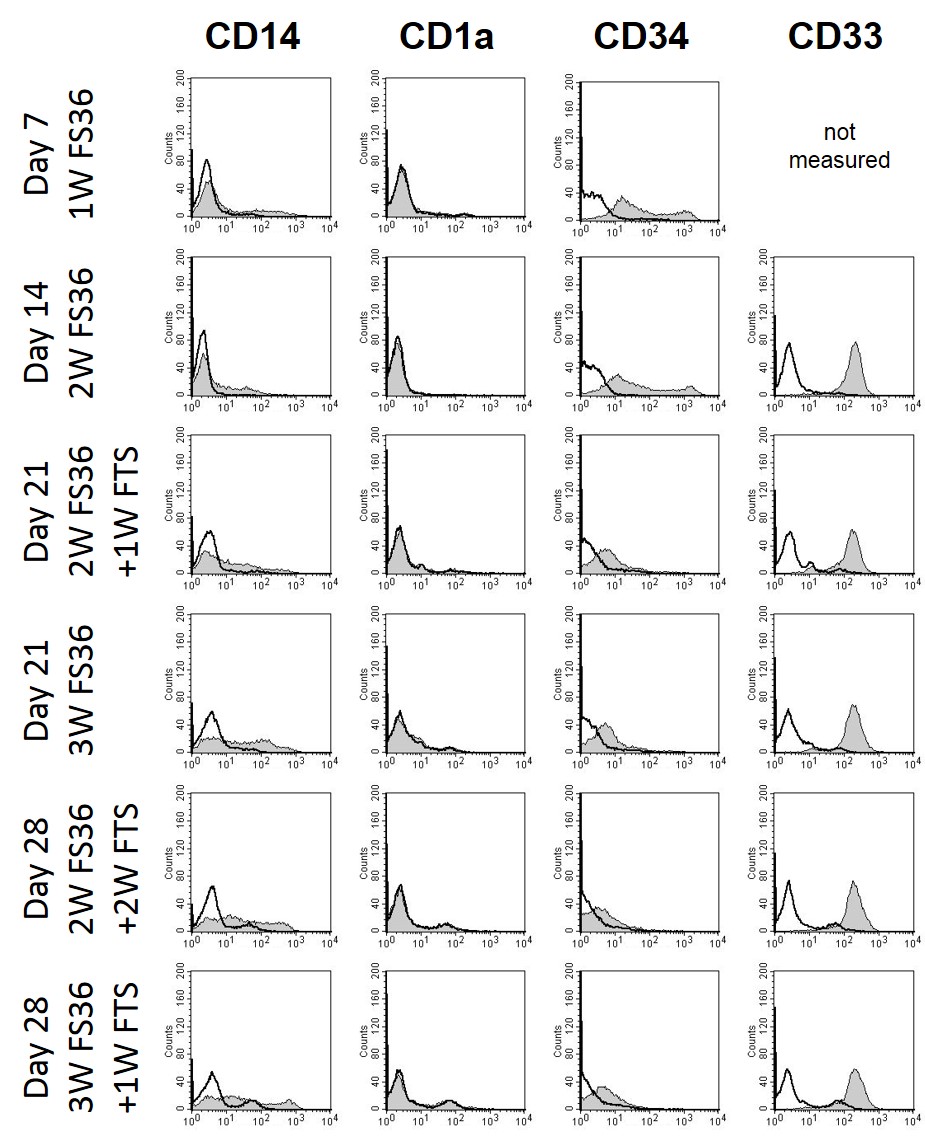


**Supplementary Figure 2S: Expansion of cord blood CD34+ progenitor cells using different cytokine cocktails.** Expression of CD14, CD1a, CD34 and CD33 was analysed by FACS at day 7, 14, 21 and 28 of expansion culture. Representative histogram overlays. (black line: isotype control, grey infill: marker expression; 1W: 1 week of culture; 2W: 2 weeks of culture; 3W: 3 weeks of culture; FS36: supplementation of Flt3-L, SCF, IL-3 and IL-6; FTS: supplementation of Flt3-L, TPO and SCF.


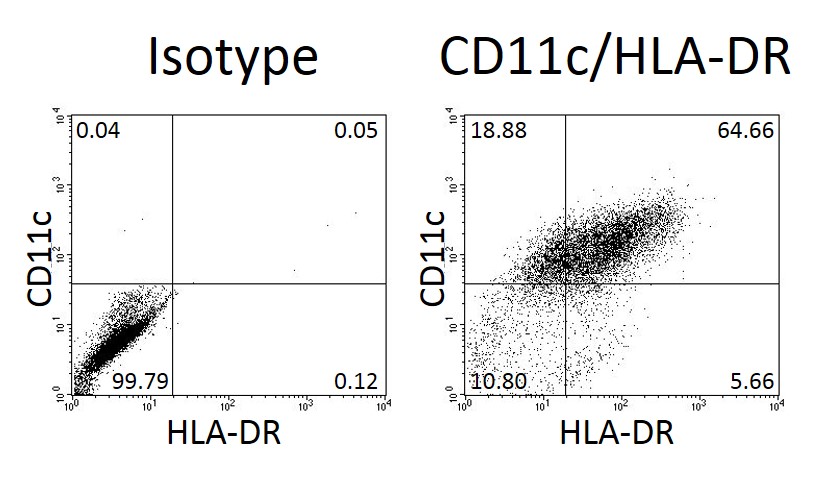


**Supplementary Figure 3S: CD11c and HLA-DR expression of neonatal DCs.** CD34+ cord blood progenitors were expanded using a cocktail of Flt-3L, SCF, IL-3 and IL-6 for 2 weeks followed by Flt-3L, TPO and SCF for 2 weeks (2W FS36+2W FTS). Subsequently, neonatal DCs were differentiated using IL-4/GM-CSF. Expression of CD11c and HLA-DR was analysed by flow cytometry and the percentage of cells indicated. Representative dot plot of 4 independent experiments.
